# Wild blueberry-derived polyphenol metabolites attenuate telomere shortening in an in vitro model of metabolic syndrome

**DOI:** 10.1007/s00394-026-04010-x

**Published:** 2026-06-06

**Authors:** Marco Rendine, Cristian Del Bo’, Patrizia Riso, Peter Møller

**Affiliations:** 1 Division of Human Nutrition, Department of Food, Environmental and Nutritional Sciences (DeFENS), Università degli Studi di Milano, Milan, Italy; 2 Section of Environmental Health, Department of Public Health, University of Copenhagen, Copenhagen, Denmark

**Keywords:** Telomere length, Blueberries, (poly)phenols, Oxidative stress, THP-1 monocytes, Metabolic syndrome

## Abstract

**Purpose:**

Metabolic syndrome (MetS) is a multifactorial disorder characterized by metabolic alterations that increase cardiovascular risk and may accelerate telomere attrition, potentially contributing to age-related diseases. Dietary (poly)phenols (PPs), including those derived from blueberries (BB), may counteract telomere shortening through their multitarget biological effects; however, additional mechanistic studies are required. This study investigated the effects of BB-derived PP metabolites, ferulic acid (FA), isoferulic acid (IA), vanillic acid (VA), and hippuric acid (HA), on telomere length (TL) in an in vitro MetS model.

**Methods:**

The MetS model was established using THP-1 monocytes exposed to free fatty acids and TNF-α. Metabolites were tested individually and in combination (MIX) at physiologically relevant concentrations (0.1–50 µM). Cytotoxicity, telomere length, and intracellular reactive oxygen species (ROS) were assessed.

**Results:**

The MetS stimulus significantly reduced TL (mean difference − 0.61; 95% CI − 0.80 to − 0.41; *p* < 0.001). Pre-treatment with FA (1 µM), VA (0.5–5 µM), and MIX (6.1 µM) significantly attenuated telomere attrition, restoring TL compared to the control (mean differences 0.30–0.37; *p* < 0.05), whereas lower concentrations of FA, IA, and HA were ineffective. ROS modulation was context-dependent: PP metabolites did not directly affect basal ROS levels but modified the response to H_2_O_2_ exposure, with VA (5 µM) and the MIX (6.1 µM) exacerbating H_2_O_2_-induced ROS generation under MetS conditions.

**Conclusion:**

These findings indicate that selected BB-derived PP metabolites could mitigate telomere shortening under metabolic stress independently of acute ROS modulation, supporting a potential role of dietary PPs in preserving genomic integrity under MetS-like conditions.

**Supplementary Information:**

The online version contains supplementary material available at 10.1007/s00394-026-04010-x.

## Introduction

Aging is a progressive, time-dependent physiological decline associated with cellular senescence, genomic instability, and increased risk of chronic, degenerative diseases [1]. It is a multifaceted process characterized by the accumulation of molecular and cellular damage over time, impairing the functional capacity of tissues and organs [2]. Epigenetic alterations, loss of proteostasis, deregulated nutrient sensing, mitochondrial dysfunction, impaired intercellular communication, and notably, telomere shortening are considered key contributors to the aging phenotype [3]. Telomeres, the repetitive non-coding DNA sequences located at the ends of linear chromosomes, act as safeguarding sequences that protect genomic integrity by preventing chromosomal degradation, end-to-end fusions, and inappropriate DNA repair activities. However, during each round of cell division, telomeres gradually shorten due to the end-replication problem, ultimately triggering replicative senescence, apoptosis, or cellular dysfunction when a critical length is reached [4–7].

Environmental exposures, including lifestyle factors, dietary patterns, psychological stress, exposure to pollutants, smoking, and sedentary behavior can accelerate telomere attrition by mechanisms involving oxidative stress and inflammation [8–10]. Shorter leukocyte telomere length (TL) has been consistently associated in observational studies with obesity, insulin resistance, and adverse lipid and glucose profiles, key components of the metabolic syndrome (MetS), as well as with cardiovascular disease and type 2 diabetes, which represent major clinical outcomes linked to this condition [11, 12]. MetS is a clinical condition defined by a cluster of metabolic abnormalities, including central obesity, hyperglycemia, dyslipidemia, and elevated blood pressure [13]. Moreover, recent studies have associated shorter TL with MetS, although the strength and consistency of this relationship vary across populations and study designs [14]. Oxidative stress induces DNA breaks at telomeric regions, which are especially sensitive to reactive oxygen species (ROS) due to their high guanine content. This telomere attrition may reflect cumulative metabolic stress and systemic low-grade inflammation, further supporting the hypothesis that telomere dynamics may serve as a biomarker of metabolic health and biological aging. As a result, telomere shortening reflects cellular aging and may contributes to the pathophysiology of age-related diseases [15].

Diet plays a pivotal role in modulating telomere dynamics, mainly through antioxidant, anti-inflammatory, and metabolic regulatory mechanisms. Among the dietary components with emerging relevance are (poly)phenol (PP) compounds, a diverse class of plant-derived secondary metabolites known for their pleiotropic biological activities [16–18]. PPs have been consistently shown to attenuate cellular aging by modulating oxidative stress pathways, enhancing mitochondrial function, inhibiting pro-inflammatory signaling, and regulating apoptotic processes [8–10]. A central aspect of PP action involves the modulation of intracellular ROS levels, which are key drivers of oxidative stress–induced telomere attrition [19]. By reducing excessive ROS production and simultaneously promoting the expression of endogenous antioxidant defenses, PPs can contribute to preserving genomic integrity and telomere stability [20].

Among PP-rich fruits, blueberries (BB) (*Vaccinium spp*) are notable for their exceptional content of anthocyanins and other flavonoids with antioxidant and anti-inflammatory properties [21, 22].

In vitro studies on acute effects of BB extracts and BB-derived PP metabolites have shown reduced intracellular ROS production levels, protection against DNA damage, and downregulation of inflammatory pathways such as NF-κB and MAPK [21, 23, 24]. In animal models, blueberry supplementation has extended lifespan in *C. elegans* [25], improved cognitive performance and reduced neuroinflammation in aged rodents [26], attenuated obesity-related oxidative stress and insulin resistance in high-fat diet-fed mice [27], and improved vascular function and endothelial health [28–30]. Moreover, BB extracts have been shown to exert protective effects in metabolically relevant tissues, including cardiomyocytes, where they counteract norepinephrine-induced oxidative damage and apoptosis [31], and intestinal cells, reducing inflammation associated with increased intestinal permeability [21].

BB-derived PP metabolites have also been reported to modulate adipocyte metabolism under lipid overload conditions. Particularly, phenolic acids such as isoferulic acid (IA), vanillic acid (VA), and syringic acids have been shown to downregulate fatty acid synthase expression and enhance antioxidant defenses, suggesting a role in regulating adipocyte metabolic function and oxidative stress [24]. In addition, anthocyanin- and phenolic acid–rich fractions from wild blueberry reduce TNF-α–induced monocyte adhesion to vascular endothelial cells, a critical early event in atherogenesis, supporting a protective role of BB bioactives against vascular inflammation and cardiovascular disease development [32].

Evidence on the potential role of PP metabolites in telomere shortening is still lacking. Given the central role of telomere attrition in aging and in the pathophysiological features of MetS, we aimed to investigate the effects of BB-derived PP metabolites on TL in a dysmetabolic and proinflammatory cell model that mimics the conditions observed in humans with MetS. As supportive mechanistic evidence, we explored whether phenolic metabolites could influence cellular responses to oxidative challenges under metabolic stress conditions, given the well-established link between oxidative stress and telomere dynamics in immune cells. To this end, we employed a human THP-1 monocyte-derived cell line exposed to a hyperlipidemic and proinflammatory medium to reproduce the metabolic stress characteristic of MetS.

## Material and methods

### THP-1 cell culture and MetS-like model

Monocytic THP-1 cells were cultured in suspension using RPMI-1640 medium supplemented with 10% fetal bovine serum (FBS) and 1% penicillin–streptomycin (P/S). Cells were maintained at a density between 3 and 9 × 10^5^ cells/mL and sub-cultured every 2–3 days. THP-1 cells were used up to a maximum of 10 passages before being discarded. Cultures were kept at 37 °C in a humidified atmosphere containing 5% CO_2_.

To mimic MetS conditions, cells were exposed for 72 h to a dysmetabolic and proinflammatory stimulus, consisting in a mixture of free fatty acids (FFAs) containing oleic acid (OA) and palmitic acid (PA) in a 2:1 ratio at a final concentration of 500 μM, combined with tumor necrosis factor-alpha (TNF-α) at 1 ng/mL, a concentration previously established to simulate low-grade inflammation [33]. The FFA combination as closely represents physiological conditions, given that OA and PA are the predominant FFAs in human plasma [24]. Similar in vitro approaches combining TNF-α and palmitate stimulation in human monocytes have been previously employed to model obesity-related inflammatory signaling and insulin resistance–associated mechanisms [34].

The FFA stock solution was prepared in ethanol and stored at − 20 °C. Prior to use, FFAs were dissolved in culture medium containing 2% bovine serum albumin (BSA) at 37 °C. The resulting FFA/BSA solution was added to the cell culture medium to reach the final 500 μM FFA concentration. Negative control cells received the same concentration of ethanol and BSA (vehicle control), with final concentrations in the medium of 0.05% ethanol and 2% BSA.

Preliminary studies were conducted to validate the impact of the MetS-like stimulus after 72 h on telomere integrity. During preliminary experiments, hydrogen peroxide (H_2_O_2_; 200 µM) was used as a positive control for TL assessment.

### Treatments with blueberry-derived (poly)phenol metabolites

Lyophilized standards of IA, VA, ferulic acid (FA), and hippuric acid (HA) were dissolved in methanol (MetOH) to prepare stock solutions at a concentration of 10 mM. Aliquots of the standards were prepared and stored at − 20 °C until use. Working solutions of each compound were freshly prepared before each experiment.

Cells were pre-treated with PP metabolites for 24 h, followed by exposure to the MetS-like stimulus (FFA + TNF-α) for 72 h (as described above).

The concentrations of PP metabolites tested were selected based on physiological plasma levels reported in human studies following blueberry consumption. These compounds were chosen because they are among the most consistently identified circulating phenolic metabolites resulting from the metabolism of dietary polyphenols in humans, including after BB intake [35–37]. Additionally, a supraphysiological concentration tenfold higher was tested to evaluate dose-dependent effects. Specifically, FA and IA were tested at 0.1 and 1 μM, and 0.5 and 5 μM, respectively; VA was tested at 0.5 and 5 μM, while HA was used at 5 and 50 μM. Two metabolite mixtures were also tested: MIX, containing the lowest concentrations of all compounds (FA 0.1 μM, IA 0.5 μM, VA 0.5 μM, HA 5 μM), and MIX × 2, containing the intermediate concentrations (FA 1 μM, IA 5 μM, VA 5 μM, HA 50 μM). The final concentration of methanol in the culture medium was kept below 0.05%, and an equivalent concentration of methanol was added to the medium of negative control cells.

### Cell viability and cytotoxicity

Cell viability was assessed using WST-1 and LDH assays. THP-1 cells were seeded in 96-well plates at a density of 2.0 × 10^4^ cells per well in 100 µL of complete medium. Cells were immediately exposed to treatments at concentrations ranging from 0.1 to 100 µM, while vehicle-treated cells served as negative controls. For the WST-1 assay, at the end of the exposure period, fresh medium containing 10% WST-1 reagent (Roche Diagnostics GmbH, Mannheim, Germany) was added to each well. Plates were incubated for 1 h at 37 °C in 5% CO_2_ before measuring absorbance at 450 nm (with 630 nm reference) using a Multiskan Ascent spectrophotometer (USA). The LDH assay was performed in parallel under the same conditions. LDH activity was measured in the cell culture supernatant using the Cytotoxicity Detection Kit (Lactate Dehydrogenase Activity, Roche) according to the manufacturer’s instructions. The LDH working solution was added to the supernatant and incubated for 30 min at room temperature before measuring absorbance at 500 nm (630 nm reference). Cells treated with 2% Triton X-100 (TX-100; Sigma-Aldrich, USA) served as positive controls. Experiments were repeated three times, with three technical replicates for each experimental condition. To be considered cytotoxic, exposures should cause more than 20% difference relative to the concurrent controls and statistically significant effects in both WST-1 and LDH assays.

### DNA extraction and telomere length analysis

Genomic DNA was extracted from THP-1 using a Quick-DNA Universal Kit (ZymoResearch, Irvine, CA, USA). After extraction, DNA concentration and purity were assessed spectrophotometrically, and only samples with a 260/280 absorbance ratio between 1.8 and 2.0 were considered suitable for analysis.

Telomere Length (TL) was quantified by quantitative real-time PCR, as previously described [38]. For the quantitative PCR the following telomere primers (written 5’to 3’) were used: CGGTTTGTTTGGGTTTGGGTTTGGGTTTGGGTTTGGGTT (forward) and GGCTTGCCTTACCCTTACCCTTACCCTTACCCTTACCCT (reverse). The primers for Single Copy Gene (36B4) were ACTGGTCTAGGACCCGAGAAG (forward) and TCAATGGTGCCTCTGGAGATT (reverse) (TAG Copenhagen A/S, Denmark). The cycles for the quantitative PCR for the TL measurement were 10 min at 95 °C, then 30 cycles at 95 °C for 15 s, followed by 54 °C for 60 s and 60 °C for 30 s. The cycling conditions for the Single Copy Gene (36B4) were 10 min at 95 °C, then 40 cycles at 95 °C for 15 s, followed by 60_C for 60 s. The qPCR was executed using 96-wells and a 7900HT Fast Real-time PCR System (Applied Biosystems, USA). The final volume of each well was 20 µl containing 2 ng of genomic DNA together with primers and 1 × SYBR Green PCR Master Mix (Applied Biosystems, USA).

All samples were analyzed in triplicates, and the results were given as a ratio (T/S):

$$ {\mathbf{Relative}} \, {\mathbf{T}}/{\mathbf{S}} \, = \, {\mathbf{1}}/\left( {{\mathbf{2}}^{{{\mathbf{Ct}}\left( {{\mathbf{telomeres}}} \right)/{\mathbf{2Ct}}({\mathbf{SCG}})}} } \right) \, = \, {\mathbf{2}}^{{ - }{{\mathbf{\Delta \Delta Ct}}}} $$

where Ct equals the number of cycles required to reach the set threshold for fluorescence accumulated in the well for either Single Copy Gene (SCG) or telomeres.

In all cellular experiments, untreated cells were used as the reference control for normalization.

### Intracellular ROS analysis

Intracellular ROS levels in THP-1 cells was measured after exposure to individual PP metabolites (FA, 0.1–1 μM; IA and VA, 0.5–5 μM; HA, 5–50 μM), a mixture of them (MIX), a MetS-like stimulus (oleic acid/palmitic acid at a 2:1 ratio, 500 μM, combined with TNFα at 1 ng/mL), and hydrogen peroxide (200 μM). All treatments were applied both individually and in combination to isolate their specific effects. Intracellular ROS production was assessed using the 2′,7′-dichlorofluorescin diacetate (DCFH-DA) assay. DCFH-DA crosses cell membranes via passive diffusion and is subsequently converted by intracellular esterases into the non-fluorescent compound DCFH. In the presence of ROS such as hydroxyl radicals, DCFH is oxidized to the fluorescent compound dichlorofluorescein (DCF). Therefore, intracellular ROS levels were quantified by measuring DCF fluorescence.

THP-1 cells (5 × 10^4^ cells/well) were seeded in 96-well plates and cultured in full cell culture medium RPMI-1640, with 10% FBS and 1% P/S) with PP metabolites (0.1–50 μM) and/or the MetS-like stimulus for 96 h at 37 °C in a humidified atmosphere containing 5% CO_2_. On the day of ROS measurement, the medium was removed and Hank’s medium with DCFH-DA was added to cells for 15 min. Subsequently, the medium was removed and new Hanks medium with 200 µM hydrogen peroxide was added to the cells, which were incubated for 3 h at 37 °C in a humidified atmosphere containing 5% CO_2_. Fluorescence was then measured after 1, 2 and 3 h using a fluorescence spectrophotometer with excitation and emission wavelengths of 485 nm and 538 nm, respectively. Three independent experiments were conducted with each treatment repeated in triplicate.

### Statistical analysis

Statistical analyses were performed using Stata 15 (StataCorp LLC, College Station, TX, USA) and GraphPad Prism 8.4.2 (GraphPad Software Inc., San Diego, CA, USA). All data were initially analyzed for outliers and normality. Outliers were identified using the ROUT method, and normality was assessed with the Shapiro–Wilk test. Regarding telomere length, differences between treatment groups were analyzed using one-way ANOVA followed by Fisher’s LSD post hoc test.

Intracellular ROS levels were analyzed using a full-factorial ANOVA with three experimental factors: treatment with PPs, MetS, and hydrogen peroxide (H_2_O_2_), and adjusted for incubation time with DCFH (1, 2, and 3 h). Statistical inference followed Yates’ analysis for 2^3^ factorial design, with non-significant interaction terms pooled into the residual variance. When significant main effects or interactions were detected, post-hoc test similar to Fisher’s LSD was applied.

For all statistical analysis, the significance was determined at a *p* value ≤ 0.05. Results are shown as mean ± standard error of the mean (SEM).

## Results

### Cytotoxicity of BB-derived PP metabolites

**Fig. 1:**
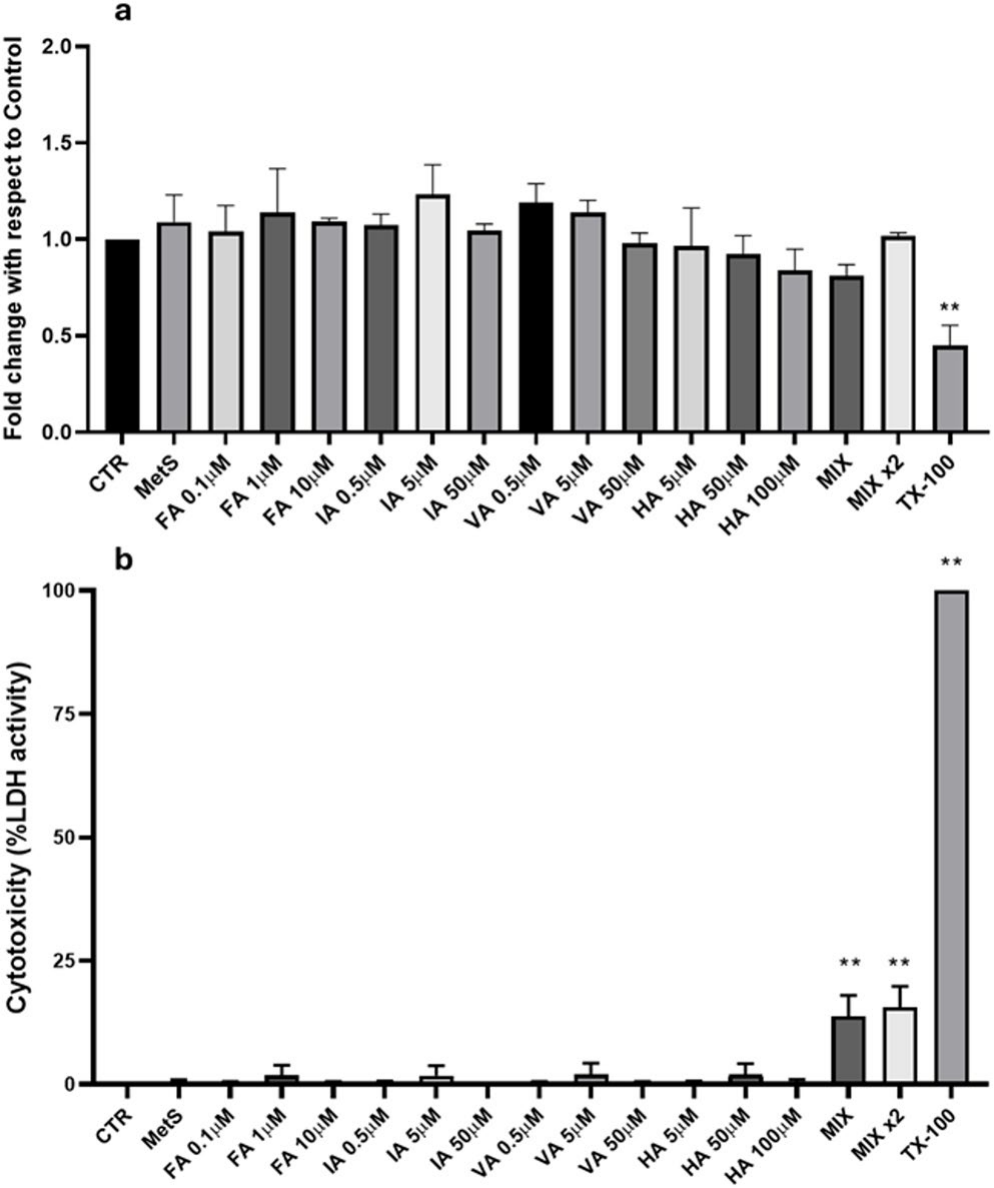
**a**, **b** Cytotoxicity measured by (**a**) the WST-1 and (**b**) LDH assays in THP-1 cells after 24 h exposure to BB-derived (poly)phenol metabolites or MetS (FFAs OA:PA 2:1, 500 µM + TNF-α 1 ng/mL). Ferulic acid (FA) was tested at 0.1, 1, and 10 μM; isoferulic acid (IA) at 0.5, 5, and 50 μM; vanillic acid (VA) at 0.5, 5, and 50 μM; and hippuric acid (HA) at 5, 50, and 100 μM. Two metabolite mixtures were also tested: MIX, containing the lowest concentrations of all compounds (FA 0.1 μM, IA 0.5 μM, VA 0.5 μM, HA 5 μM), and MIX × 2, containing the intermediate concentrations (FA 1 μM, IA 5 μM, VA 5 μM, HA 50 μM). Cells treated with 2% Triton X-100 were used as positive control. Data are presented as mean ± SEM (n = 3). Significant differences between groups are indicated by different letters (a, b; *p* < 0.05)

The potential cytotoxicity of BB-derived PP metabolites on THP-1 cells was assessed using WST-1 and LDH assays (Fig. 1a, b). Metabolic activity is shown in Fig. 1a and is presented as a percentage relative to the negative control following 24 h of exposure to PP metabolites. No statistically significant effects on metabolic activity were observed for any of the PP metabolites tested. Similarly, the MetS-like stimulus (FFA + TNFα) did not significantly affect cellular metabolic activity. In contrast, cells treated with the positive control (2% Triton X-100, TX-100) showed a significant reduction (−55%) in metabolic activity (*p* < 0.01), indicating cell death.

Membrane integrity was evaluated using the LDH assay which results, expressed as percentage of positive control (2% TX-100), are reported in Fig. 1b. LDH release was consistent with the WST-1 results, showing no statistically significant increase compared to the negative control after 24-h exposure to any of the tested PP metabolites, except for the mixture at both low (MIX) and high (MIX × 2) concentration, which slightly increased LDH activity (approximately 15% compared to the negative control). For this reason, only the MIX reflecting physiologically relevant concentrations was included in the subsequent experiments.

### MetS-induced THP-1 cells show enhanced telomere shortening

This section reports the results of the experiments conducted to validate the in vitro MetS-like model by assessing its effects on TL in THP-1 cells (Supplementary 1, Fig. S1).

Following MetS treatment, THP-1 cells exhibited a statistically significant reduction in TL compared to the untreated control (mean difference: −0.61; 95% CI −0.80 to −0.41; *p* < 0.001). In these preliminary experiments, cells were also treated with H_2_O_2_ (200 nM) as a positive control. H_2_O_2_ exposure similarly led to a significant decrease in TL (mean difference: −0.48; 95% CI −0.68 to −0.29; *p* < 0.001).

The effect of MetS was comparable to that of H_2_O_2_, with no statistically significant difference observed between the two conditions (mean difference: −0.12; 95% CI −0.31 to 0.02; *p* > 0.05).

### BB-derived PP metabolites mitigate telomere shortening in THP-1 Cells

**Fig. 2:**
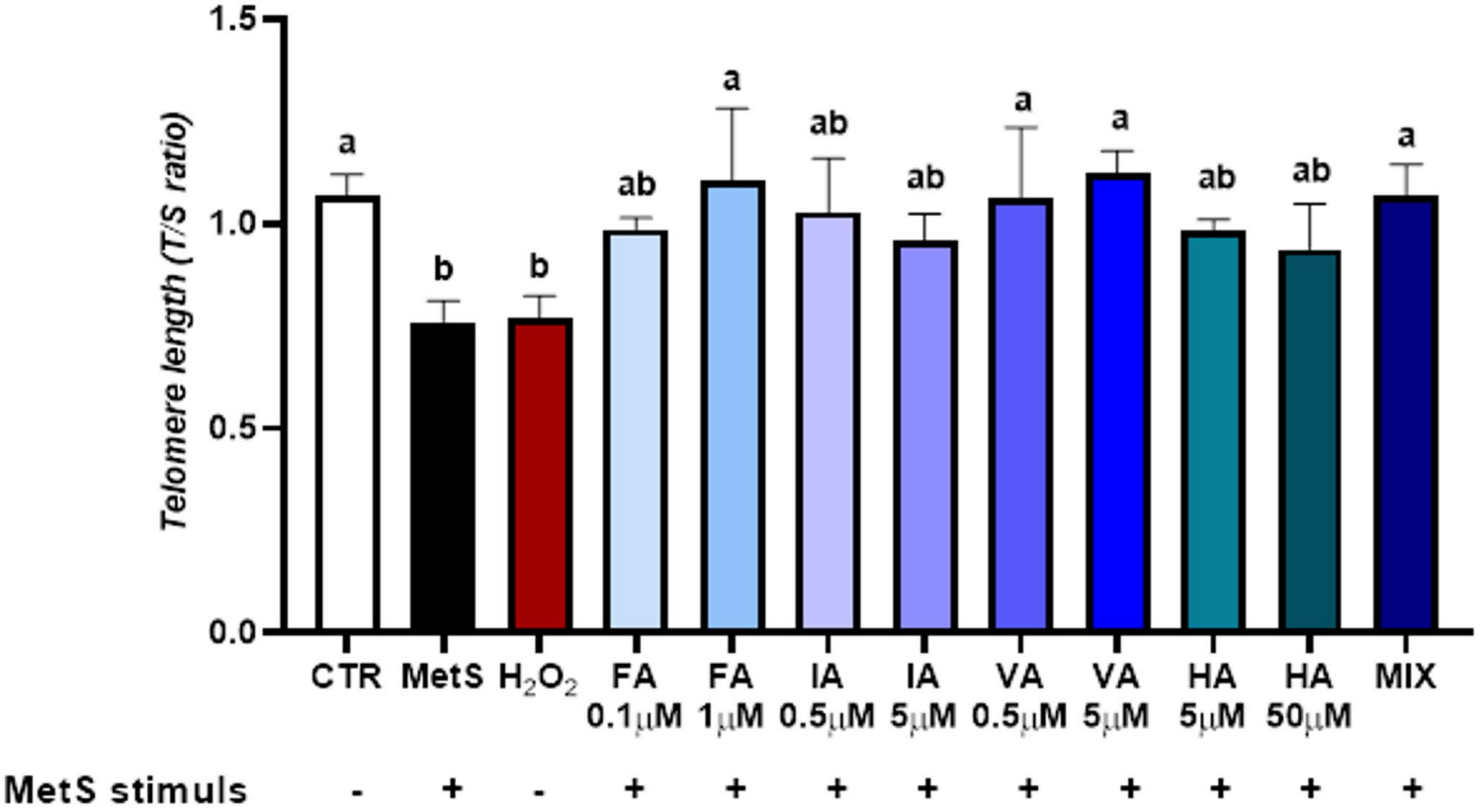
Preventive effects of BB-derived (poly)phenol metabolites on telomere length (TL) in THP-1 cells. Cells were pre-treated for 24 h with individual metabolites, including ferulic acid (FA), isoferulic acid (IA), vanillic acid (VA), and hippuric acid (HA), provided at physiologically relevant and supraphysiological concentrations, or with a mixture (MIX) containing all compounds at their physiological concentrations. After pre-treatment with metabolites, cells were exposed to a MetS-like stimulus (FFAs OA:PA 2:1, 500 µM + TNF-α 1 ng/mL) for 72 h. Untreated control (CTR) cells, MetS and H_2_O_2_ received only vehicle (2% BSA and 0.05% MeOH), MetS-like stimulus or H_2_O_2_ (200 µM) for 72 h, respectively. TL was assessed by qPCR and expressed as relative T/S ratio normalized to the control. Data are presented as mean ± SEM (n = 3). Significant differences between groups are indicated by different letters (a, b; *p* < 0.01)

The results on protective effects of BB-derived PP metabolites against telomere shortening are shown in Fig. 2. All compounds were administered at both physiologically relevant and supraphysiological concentrations. The MIX represented the combination of all four metabolites at their respective physiological concentrations.

Pre-treatment with FA at 1 µM significantly attenuated telomere shortening compared to cells exposed to the MetS-like stimulus alone (mean difference: 0.35; 95% CI 0.06 to 0.63; *p* < 0.05), restoring TL to levels comparable to untreated control. Similarly, VA at both 0.5 µM and 5 µM significantly prevented telomere attrition induced by the MetS-like conditions (mean differences: 0.30 [95% CI 0.02–0.59; *p* < 0.05] and 0.37 [95% CI 0.08–0.65; *p* < 0.05], respectively). In addition, the MIX significantly preserved TL compared to the MetS-induced cells (mean difference: 0.31; 95% CI 0.02–0.59; *p* < 0.05).

Conversely, pre-treatments with FA, IA and HA at 0.1 µM did not significantly affect MetS-induced telomere shortening.

### Effects of BB-derived PP metabolites on intracellular ROS production

**Fig. 3:**
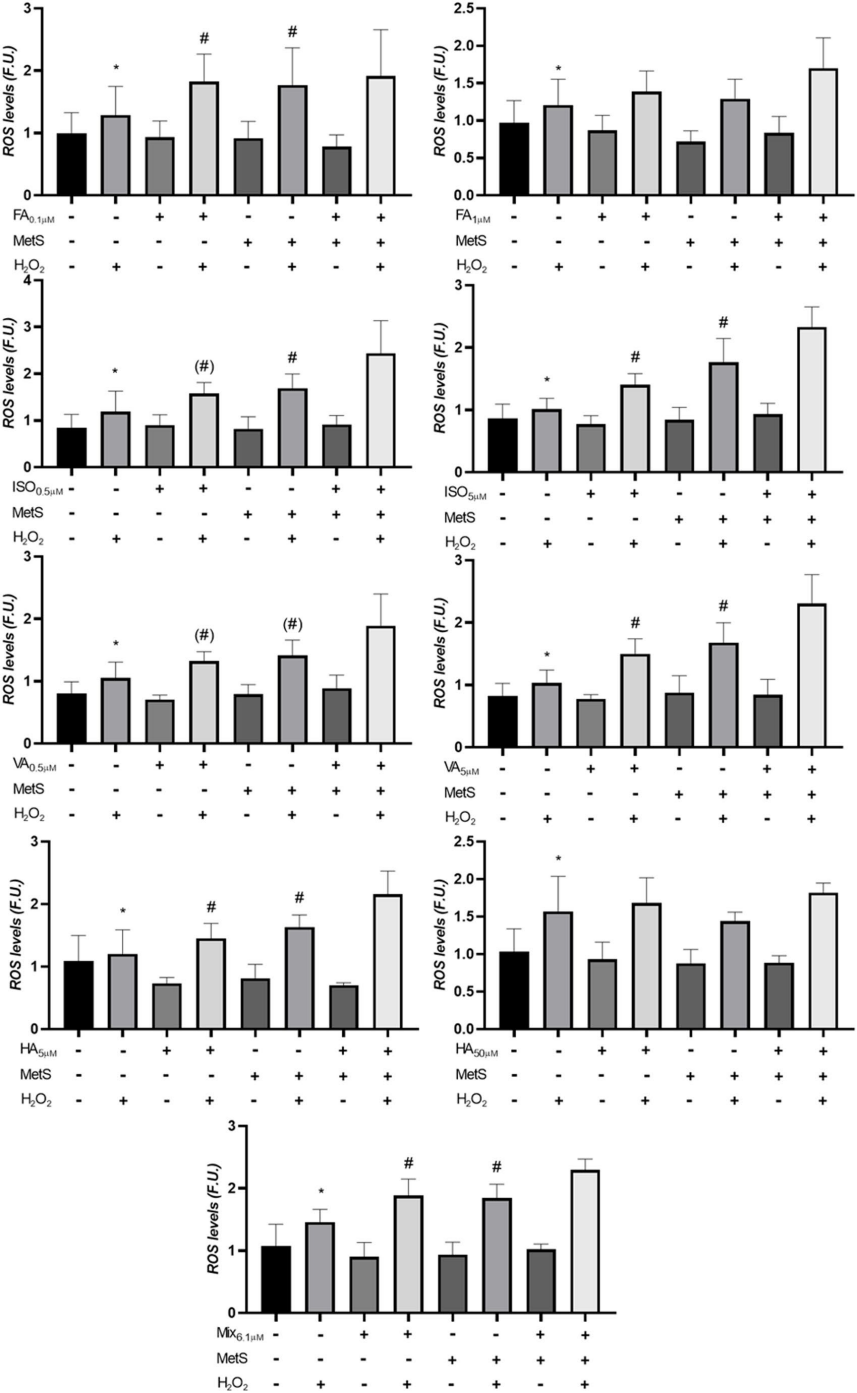
**a**–**g** Effects of BB-derived (poly)phenol metabolites on background and H_2_O_2_-induced intracellular ROS production in THP-1 cells. Cells were pre-treated for 24 h with individual (poly)phenol metabolites, including (**a**, **b**) ferulic acid (FA), (**c**, **d**) isoferulic acid (IA), (**e**, **f**) vanillic acid (VA), and (**g**, **h**) hippuric acid (HA). Single compounds were provided at physiologically relevant and supraphysiological concentrations. (i) the mixture (MIX) containing all compounds at their physiological concentrations. After pre-treatment, cells were exposed to a MetS-like stimulus (FFAs OA:PA 2:1, 500 µM + TNF-α 1 ng/mL) for 72 h. Untreated control cells received only vehicle (2% BSA and 0.05% MeOH). Data are presented as mean of three DCFH incubation times (1, 2 and 3 h) ± SEM (n = 3). Significant differences between groups are indicated by different symbols (* indicates single-factor effects; # indicates interactions; *p* < 0.05)

The effect of BB-derived PP metabolites on background and H_2_O_2_-induced intracellular ROS levels was investigated by quantifying intracellular ROS production. H_2_O_2_ does not react with DCFH; however, in cells H_2_O_2_ is converted to reactive species such as hydroxyl radicals that react with DCFH. In addition, as DCFH reacts with as number of other radical species, the DCFH assay is considered to measure the rate of ROS production during the incubation rather than an assay for detection of specific types of ROS in cells. ROS levels increased with DCFH incubation time; however, this increase was independent of the type of treatment (PPs, MetS, or H_2_O_2_). Therefore, results are reported as the mean of the three DCFH incubation times (Fig. 3a–g). Complete data for each incubation time (1–3 h) and for each compound are provided in Supplementary file 1 (Tables S1–S8). As expected, exposure to H_2_O_2_ increased ROS levels in all experiments (*p* < 0.05). The results related to PP metabolites and interaction with other treatments (i.e. H_2_O_2_ and/or MetS) are summarized in Table 1. They indicate a relatively complex relationship between concentration-dependent effects of PPs and MetS on H_2_O_2_-induced ROS levels. Overall, MetS and PP metabolites did not have direct effect on ROS levels, whereas they affect ROS levels produced by H_2_O_2_. Moreover, this interaction depends on the concentration of PP metabolites. Physiological concentrations of FA and HA, and supraphysiological concentrations of IA and VA, showed statistically significantly higher ROS levels after H_2_O_2_ exposure (i.e., interactions between PP metabolites and H_2_O_2_, and MetS and H_2_O_2_). At high FA and HA concentration, there was no effect of interactions with PP metabolites and MetS. At low IA and VA concentrations, MetS treatment increased H_2_O_2_-generated ROS level (*i.e.,* interaction between MetS and H_2_O_2_). In general, the results indicate that FA and VA produce one kind of effect on ROS production, whereas IA and HA produce a different effect. The mixture of PP metabolites (MIX) produced the same effect as the low concentration of FA/HA or high concentration of IA/VA, i.e. a potentiation of H_2_O_2_-generated ROS production (*p* < 0.05 for interaction between MetS and H_2_O_2_, and PP metabolites and H_2_O_2_).

**Table 1:** Summary of the effects of wild blueberry (poly)phenol (PP) metabolites alone or in combination with hydrogen peroxide (H_2_O_2_) and/or metabolic syndrome (MetS) on the evaluated background or H_2_O_2_-generated intracellular reactive oxygen radical (ROS) level

Treatment	H_2_O_2_/MetS	PP/H_2_O_2_	H_2_O_2_
FA 0.1 µM	↑	**↑**	↑
FA 1 µM	–	–	↑
IA 0.5 µM	↑	(↑)	↑
IA 5 µM	**↑**	**↑**	↑
VA 0.5 µM	↑	(↑)	↑
VA 5 µM	**↑**	**↑**	↑
HA 5 µM	**↑**	**↑**	↑
HA 50 µM	–	–	↑
MIX 6.1 µM	↑	**↑**	↑

Ferulic acid (FA), isoferulic acid (IA), vanillic acid (VA), and hippuric acid (HA), mixture containing all compounds (MIX). ↑ indicates an increase in ROS production, while (↑) indicates an increase, which is of borderline statistical significance in overall ANOVA and statistically significant in post-hoc test.—indicates a lack of statistical significance. Normal and bold arrows represent effects that are modestly (*P* < 0.05) and highly (*P* < 0.01) statistically significant, respectively

## Discussion

This study aimed to evaluate, for the first time, the potential protective effects of BB-derived PP metabolites, specifically FA, IA, VA, and HA, and a mixture of them (MIX), on telomere shortening in an in vitro monocyte MetS-like model. Furthermore, we investigated whether these metabolites could mechanistically attenuate ROS production in hydrogen peroxide(H_2_O_2_)-exposed cells. Through this integrated approach, we explored the impact of PP metabolites on telomere biology, a hallmark of aging and age-related chronic diseases, while also clarifying the contribution of ROS to this process.

The selected BB-derived metabolites (FA, IA, VA, and HA) are among the main phase II and microbial metabolites found in blood after BB consumption [37]. The in vitro concentrations were selected to mimic physiologically as well as supraphysiologically achievable levels based on human pharmacokinetic data. The physiological concentrations used in our in vitro model reflect the plasma peak levels of these metabolites observed in previous studies after the consumption of BB [39]. We included supraphysiological concentrations to explore potential dose-dependent effects and to assess the maximal biological response of each compound. Furthermore, we tested a metabolite mixture designed to mimic their combined presence in the human blood following BB consumption.

Our in vitro data showed that THP-1 monocytes exposed to TNF-α + FFAs, mimicking the chronic low-grade inflammation occurring in MetS, triggered telomere shortening. This is consistent with human data showing accelerated telomere attrition in MetS patients [14]. It is important to emphasize that this approach does not aim to reproduce MetS as a systemic disease, but rather to model a simplified state of metabolic and inflammatory stress characterized by elevated FFAs and low-grade inflammatory signaling. In this cell model, treatment with VA (1 µM), FA (0.5 and 5 µM), and the metabolite MIX (physiological concentration) effectively attenuated telomere loss. These results align with literature on dietary PPs modulating telomere length. For instance, resveratrol upregulates telomerase activity and reduces DNA damage in endothelial progenitor cells [40]. Moreover, epigallocatechin-3-gallate (EGCG, 50–100 mg/kg) and quercetin (100 mg/kg) have been shown to prevent telomere shortening and the loss of telomere repeat-binding factor 2 (TRF2) in the hypertrophic myocardium of rats [41].

Interestingly, evidence showed that the effects of the green tea-derived EGCG on TL can vary depending on the cell type, showing opposite outcomes in immortalized versus primary cells. Treatment with EGCG (20–200 µM) led to telomere shortening and reduced telomerase activity in the immortalized Caco-2 cell line, whereas in primary fibroblasts it was associated with relatively longer telomeres and increased methylation at six CpG sites in the promoter region of human Telomerase Reverse Transcriptase (hTERT) [42]. In addition to PPs, other bioactives have been investigated for their ability to influence telomere dynamics. For instance, palm-derived γ-tocotrienol has demonstrated protective effects against oxidative stress-induced cellular ageing in primary human fibroblasts obtained from young (21y) and old donors (68y). In fibroblasts from both young and old subjects, pretreatment with γ-tocotrienol prevented telomere shortening and maintained telomerase activity, underscoring its potential to counteract stress-induced telomere attrition in primary, non-immortalized cells [43].

While evidence from human studies remains limited, some investigations suggest a positive association between PP-rich diets and telomere length. Observational studies have associated polyphenol-rich dietary patterns with longer telomeres. Adherence to the Mediterranean diet, rich in plant-based foods and polyphenols, has been linked to longer TL in elderly individuals [44]. Similarly, higher intakes of specific flavonoid subclasses, such as flavan-3-ols and anthocyanins, have been positively associated with TL in large population-based cohorts including NHANES and the Nurses’ Health Study [45, 46]. Additionally, Gong et al. (2018) reported a positive correlation between TL and a vegetable-rich dietary pattern characterized by increased intake of fruits, leafy vegetables, and tea, all established sources of dietary PPs [47]. However, randomized controlled trials directly investigating the impact of PP-rich foods on telomere-related outcomes remain scarce. Notably, a recent randomized controlled trial demonstrated a significant increase (~ 25%) in telomerase activity in CD8 + T lymphocytes of healthy volunteers following a 5-day intervention with cooked leaves of *Brassica carinata*, highlighting the potential for rapid modulation of telomere biology through dietary components [47].

PPs, including those derived from BB, can modulate various metabolic pathways closely related to TL and cellular aging, particularly oxidative stress and inflammation [24, 48]. Oxidative stress, through the high level of ROS, can directly damage telomeric DNA, which is especially susceptible due to its high guanine content. This leads to accelerated telomere shortening and promotes cellular senescence [49, 50]. PPs (e.g., quercetin, resveratrol, curcumin) exhibit strong antioxidant and anti-inflammatory properties. These molecules have been shown to reduce ROS levels, inhibit NF-κB signaling, and modulate key enzymes involved in redox balance [20, 51].

Therefore, we hypothesized that PPs exert a protective role against telomere shortening through modulation of cellular oxidative status [20, 52]. To this end, we investigated the effects of PP metabolites on intracellular ROS levels in THP-1 monocytes exposed to metabolic stress conditions, with or without an additional oxidative challenge induced by hydrogen peroxide. It should be noted that intracellular ROS measurements were used as a general indicator of changes in cellular oxidative status, rather than to infer specific ROS-generating or scavenging mechanisms. Our factorial analysis demonstrated that neither PPs nor the MetS stimulus directly altered basal ROS levels. However, both factors modulated the response to H_2_O_2_, revealing a complex pattern of interactions that depended on metabolite concentration. As expected, exposure to H_2_O_2_ alone significantly increased ROS levels across all experiments, confirming its role as a potent oxidative stressor. It is interesting to note that PP metabolites and MetS had the same effect on ROS levels, i.e. increased vulnerability of cells to a sudden bolus exposure to H_2_O_2_. Notable, there was no interaction between PP metabolites, MetS and H_2_O_2_, but higher ROS levels after H_2_O_2_ exposure in combination with either PP metabolites or MetS (i.e. independent interactions between PPs and H_2_O_2_ and between MetS and H_2_O_2_). It should be noted that the cell culture period was relatively long to assess effects in conditions similar to the TL studies. Thus, there is a possibility of adaptation of the cellular response to PP metabolites and MetS, which contrasts the cell culture condition of acute effects usually carried out in in vitro studies. It is possible that MetS-like conditions lead to a depletion of intracellular antioxidant resources that may be saturated by a sudden exposure to oxidants such as H_2_O_2_. In a similar way, PP metabolites may modulate cellular oxidative responsiveness in a manner that counterbalances basal ROS production while potentially increasing susceptibility to acute oxidant challenges.

Notably, VA was associated with a stronger increase in H_2_O_2_-induced ROS levels, at both physiological and supraphysiological concentrations. A similar potentiating effect was observed under MetS-like conditions, which alone had no impact on ROS levels but significantly amplified the oxidative response when combined with H_2_O_2_. In contrast, FA and HA at higher concentrations did not significantly interact with either H_2_O_2_ or MetS, while IA and VA showed a tendency to promote ROS generation at higher concentrations. Interestingly, the mixture of PPs mirrored the effects of low FA and HA or high IA and VA concentrations, resulting in an overall enhancement of H_2_O_2_-induced oxidative stress.

These findings resonate with the dual antioxidant–pro-oxidant nature of PPs, which has been widely reported in the literature. It is well established that the redox behavior of phenolic compounds depends on concentration, cellular context, and the presence of transition metals or pre-existing oxidative stimuli [53, 54]. For instance, Magiera et al. demonstrated that VA reduced oxidative stress in human neutrophils and increased plasma antioxidant capacity, supporting its role as an antioxidant under physiological conditions [39]. Similarly, Amin et al. showed that VA reduced oxidative stress and neuroinflammation in a mouse model through activation of the Nrf2/HO-1 pathway, and protected HT22 hippocampal cells by lowering ROS and improving cell viability [55]. However, in our H_2_O_2_-induced stress model, VA behaved as a pro-oxidant, highlighting the context-dependent nature of the observed oxidative response: it may exert protective effects under mild or chronic oxidative stress, but contribute to ROS amplification under acute oxidative insults.

In our study, the physiological concentration showed pro-oxidant effects under acute H_2_O_2_ stress. In other studies, FA predominantly exhibits antioxidant and vasoprotective properties. FA inhibits angiotensin II–induced ROS generation and VSMC proliferation by suppressing ERK1/2 and JNK signaling [56]. Likewise, Lin et al. demonstrated that FA enhanced cell viability, scavenged free radicals, increased SOD activity, and reduced lipid peroxidation, apoptosis, and MAPK activation in hypoxia-stressed PC12 cells [57]. Regarding IA, although it has been shown to possess strong multi-target antioxidant activity [58], it exhibited a pro-oxidant tendency at high concentrations in our H_2_O_2_-induced THP-1 model, suggesting that the oxidative response observed in this model is influenced by the acute oxidative challenge.

In contrast, HA has been previously reported to display context-dependent pro-oxidant behavior. In vitro and in vivo studies demonstrated that HA disrupts redox homeostasis by suppressing NRF2-driven antioxidant defenses, leading to ROS accumulation and promoting fibrosis in kidney cells [59]. Similarly, HA was identified as a microbiota-derived metabolite that mediates excessive ROS accumulation in skin melanocytes via interaction with ROS-related targets such as NOS2 and MAPK14 [60]. These findings are consistent with our data, suggesting that HA can contribute to oxidative stress under pathological conditions and reinforcing its dual role as a ROS modulator depending on cellular context.

Overall, these findings should be interpreted as descriptive of changes in cellular oxidative responsiveness under the experimental conditions tested, rather than as direct mechanistic evidence of antioxidant or pro-oxidant activity of the metabolites tested.

In addition to pre-clinical evidence, several RCTs have reported a positive effect of BB and its PP against oxidative stress, including an increase in the levels of reduced glutathione (GSH) [61], a decrease in NADPH oxidase activity [35], as well as reductions in plasma oxidative stress and ROS generation potential [62]. Similarly to the effects on TL observed in our study, the antioxidant potential of BB has also been demonstrated in isolated cells, showing a decrease in free radical levels in whole blood and isolated monocytes [63], as well as a reduction in hydrogen peroxide-induced DNA damage in blood cells following BB interventions [64].

Altogether, our results highlight the complexity of BB-derived PP-mediated redox modulation and its relationship to other adverse cellular outcomes such as telomere shortening. This antioxidant-oxidant duality is consistent with the broader literature on PPs, which emphasizes that their function is not universally antioxidant but instead depends on the concentration, cellular environment, and oxidative context [53, 54, 65, 66]. Finally, these results should be interpreted within the context of a simplified in vitro model designed to explore potential adaptive cellular responses to metabolic and inflammatory stress induced by PP-derived metabolites, rather than as a direct representation of in vivo metabolic syndrome physiology.

## Conclusion

This study demonstrates that BB-derived PP metabolites, specifically FA, IA, VA and HA, can attenuate telomere shortening in THP-1 monocytes under MetS-like stress conditions. Mainly, BB-derived metabolites effectively preserved telomere length, even in the absence of consistent acute ROS reduction, suggesting that telomere protection may involve long-term modulation of stress-response pathways (e.g., Nrf2 activation, NF-κB and MAPK suppression, and potential direct effects on telomerase activity).

Overall, BB-derived metabolites may support telomere integrity and cellular resilience through mechanisms beyond direct ROS scavenging, potentially contributing to the prevention of oxidative stress-induced cellular aging. Future studies examining enzymatic antioxidant pathways, mitochondrial redox status, and telomerase activity will be essential to clarify the role of these metabolites and their potential to mitigate metabolic and oxidative disorders. Finally, the demonstration of such effects in humans will be crucial for determining the clinical relevance of the results obtained and thus, the contribution of BB in the context of metabolic syndrome and aging-related processes.

## Supplementary Information

Below is the link to the electronic supplementary material.

Supplementary Material 1

## Data Availability

Raw data are available from the corresponding author upon request.
