# Supplementary material for: Wild blueberry-derived polyphenol metabolites attenuate telomere shortening in an in vitro model of metabolic syndrome
Source: Eur J Nutr. 2026 Jun 6;65(4):157. doi: 10.1007/s00394-026-04010-x (PMC13242478; doi:10.1007/s00394-026-04010-x)
Supplement: Supplementary file 1 — Supplementary Material 1 [file 394_2026_4010_MOESM1_ESM.docx]

**Table S1.** Intracellular ROS production in cells after exposure to 0.1 µM FA

| **FA (0.1 µM)** | **MetS** | **H_2_O_2_** | **Time (1 h)** | **Time (2 h)** | **Time (3 h)** | **Average^a^** |
| --- | --- | --- | --- | --- | --- | --- |
| *-* | *-* | *-* | 0.89 | 1.00 | 1.08 | ***0.67 (0.51, 0.84)*** |
| *-* | *-* | *+* | 1.11 | 1.27 | 1.47 | ***0.82 (0.65, 0.98)*** |
| *+* | *-* | *-* | 0.83 | 0.95 | 1.03 | *0.69 (0.52, 0.85)* |
| *+* | *-* | *+* | 1.53 | 1.76 | 2.18 | *1.24 (1.07, 1.40)** |
| *-* | *+* | *-* | 0.84 | 0.94 | 0.99 | **0.62 (0.46, 0.79)** |
| *-* | *+* | *+* | 1.57 | 1.78 | 1.96 | **1.24 (1.08, 1.40)**** |
| *+* | *+* | *-* | 0.67 | 0.80 | 0.88 | 0.65 (0.49, 0.81) |
| *+* | *+* | *+* | 1.65 | 1.93 | 2.18 | 1.45 (1.29, 1.61) |
|  |  |  |  |  |  |  |
| **Effect size of MetS and H_2_O_2_ interaction (P_interaction_ = 0.019)** | | | | | | |
| MetS | -0.04 (95% CI: -0.20, 0.12) | | | | | |
| H_2_O_2_ | 0.35 (95% CI: 0.19, 0.51) | | | | | |
| Interaction | 0.32 (95% CI: 0.16, 0.48) | | | | | |
| **Effect size of FA and H_2_O_2_ interaction (P_interaction_ = 0.003)** | | | | | | |
| FA | 0.02 (95% CI: -0.14, 0.18) | | | | | |
| H_2_O_2_ | 0.38 (95% CI: 0.22, 0.54) | | | | | |
| Interaction | 0.31 (95%CI: 0.15, 0.48) | | | | | |

^a^Values are mean (95% confidence interval) of intracellular ROS production in fluorescence units, adjusted for time. Bold text cover groups involved in the MetS and H_2_O_2_ interaction. Italic text cover groups involved in the FA and H_2_O_2_ interaction.

**Table S2.** Intracellular ROS production in cells after exposure to 1 µM FA

| **FA (1 µM)** | **MetS** | **H_2_O_2_** | **Time (1 h)** | **Time (2 h)** | **Time (3 h)** | **Average^a^** |
| --- | --- | --- | --- | --- | --- | --- |
| *-* | *-* | *-* | 0.88 | 0.98 | 1.05 | 1.30 (0.86, 1.73) |
| *-* | *-* | *+* | 1.09 | 1.21 | 1.33 | **1.68 (1.24, 2.11)^a^** |
| *+* | *-* | *-* | 0.78 | 0.87 | 0.95 | 1.11 (0.68, 1.55) |
| *+* | *-* | *+* | 1.23 | 1.39 | 1.53 | **1.97 (1.53, 2.41)^a^** |
| *-* | *+* | *-* | 0.65 | 0.73 | 0.78 | 1.02 (0.58, 1.45) |
| *-* | *+* | *+* | 1.14 | 1.30 | 1.44 | **1.82 (1.39, 2.26)^a^** |
| *+* | *+* | *-* | 0.72 | 0.85 | 0.95 | 0.97 (0.54, 1.41) |
| *+* | *+* | *+* | 1.50 | 1.71 | 1.89 | **2.14 (1.73, 2.61)^a^** |
|  |  |  |  |  |  |  |
| **Effect size of MetS and H_2_O_2_ interaction (P_interaction_ = 0.21)** | | | | | | |
| MetS | -0.21 (95% CI: -0.64, 0.22) | | | | | |
| H_2_O_2_ | 0.62 (95% CI: 0.19, 1.06) | | | | | |
| Interaction | 0.17 (95% CI: -0.26, 0.60) | | | | | |
| **Effect size of FA and H_2_O_2_ interaction (P_interaction_ = 0.16)** | | | | | | |
| FA | -0.11 (95% CI: -0.54, 0.32) | | | | | |
| H_2_O_2_ | 0.59 (95% CI: 0.17, 1.02) | | | | | |
| Interaction | 0.32 (95% CI: -0.11, 0.75) | | | | | |

^a^ Values are mean (95% confidence interval) of intracellular ROS production in fluorescence units, adjusted for time. Bold text cover groups with single-factor effect of H_2_O_2_.

**Table S3.** Intracellular ROS production in cells after exposure to 0.5 µM IA

| **IA (0.5 µM)** | **MetS** | **H_2_O_2_** | **Time (1 h)** | **Time (2 h)** | **Time (3 h)** | **Average^a^** |
| --- | --- | --- | --- | --- | --- | --- |
| *-* | *-* | *-* | 0.78 | 0.86 | 0.92 | ***0.85 (0.48, 1.23)*** |
| *-* | *-* | *+* | 1.06 | 1.19 | 1.31 | ***1.19 (0.81, 1.56)*** |
| *+* | *-* | *-* | 0.82 | 0.91 | 0.98 | *0.90 (0.53, 1.28)* |
| *+* | *-* | *+* | 1.41 | 1.58 | 1.73 | *1.58 (1.20, 1.95)* |
| *-* | *+* | *-* | 0.75 | 0.83 | 0.90 | **0.82 (0.45, 1.20)** |
| *-* | *+* | *+* | 1.49 | 1.72 | 1.86 | **1.69 (1.31, 2.06)*** |
| *+* | *+* | *-* | 0.81 | 0.94 | 1.02 | 0.92 (0.55, 1.30) |
| *+* | *+* | *+* | 2.14 | 2.44 | 2.74 | 2.44 (2.07, 2.82) |
|  |  |  |  |  |  |  |
| **Effect size of MetS and H_2_O_2_ interaction (P_interaction_ = 0.011)** | | | | | | |
| MetS | -0.00 (95% CI: -0.37, 0.37) | | | | | |
| H_2_O_2_ | 0.51 (95% CI: 0.13, 0.88) | | | | | |
| Interaction | 0.68 (95% CI: 0.31, 1.05) | | | | | |
| **Effect size of IA and H_2_O_2_ interaction (P_interaction_ = 0.063)^b^** | | | | | | |
| IA | 0.07 (95% CI: -0.30, 0.44) | | | | | |
| H_2_O_2_ | 0.60 (95% CI: 0.23, 0.97) | | | | | |
| Interaction | 0.57 (95% CI: 0.20, 0.94) | | | | | |

^a^Values are mean (95% confidence interval) of intracellular ROS production in fluorescence units, adjusted for time. ^b^Estimation of effect size is based on host-hoc test; this is statistically significant, although it should be emphasized that the overall ANOVA only showed a difference of borderline statistical significance for the interaction. Bold text cover groups involved in the MetS and H_2_O_2_ interaction. Italic text cover groups involved in the IA and H_2_O_2_ interaction.

**Table S4.** Intracellular ROS production in cells after exposure to 5 µM IA

| **IA (5 µM)** | **MetS** | **H_2_O_2_** | **Time (1 h)** | **Time (2 h)** | **Time (3 h)** | **Average^a^** |
| --- | --- | --- | --- | --- | --- | --- |
| *-* | *-* | *-* | 0.77 | 0.87 | 0.95 | ***0.86 (0.62, 1.11)*** |
| *-* | *-* | *+* | 0.91 | 1.02 | 1.11 | ***1.01 (0.77, 1.26)*** |
| *+* | *-* | *-* | 0.69 | 0.77 | 0.84 | *0.77 (0.52, 1.01)* |
| *+* | *-* | *+* | 1.24 | 1.41 | 1.55 | *1.40 (1.16, 1.65)*** |
| *-* | *+* | *-* | 0.67 | 0.88 | 0.97 | **0.840.60, 1.09)** |
| *-* | *+* | *+* | 1.56 | 1.79 | 1.95 | **1.77 (1.52, 2.01)***** |
| *+* | *+* | *-* | 0.80 | 0.95 | 1.05 | 0.93 (0.69, 1.18) |
| *+* | *+* | *+* | 2.05 | 2.34 | 2.60 | 2.32 (2.08, 2.58) |
|  |  |  |  |  |  |  |
| **Effect size of MetS and H_2_O_2_ interaction (P_interaction_ < 0.001)** | | | | | | |
| MetS | -0.00 (95% CI: -0.24, 0.24) | | | | | |
| H_2_O_2_ | 0.54 (95% CI: 0.29, 0.78) | | | | | |
| Interaction | 0.48 (95%CI: 0.23, 0.72) | | | | | |
| **Effect size of IA and H_2_O_2_ interaction (P_interaction_ = 0.008)** | | | | | | |
| IA | 0.07 (95% CI: -0.17, 0.31) | | | | | |
| H_2_O_2_ | 0.39 (95% CI: 0.15, 0.63) | | | | | |
| Interaction | 0.84 (95%CI: 0.60, 1.08) | | | | | |

^a^Values are mean (95% confidence interval) of intracellular ROS production in fluorescence units, adjusted for time. Bold text cover groups involved in the MetS and H_2_O_2_ interaction. Italic text cover groups involved in the IA and H_2_O_2_ interaction.

**Table S5.** Intracellular ROS production in cells after exposure to 0.5 µM VA

| **VA (0.5 µM)** | **MetS** | **H_2_O_2_** | **Time (1 h)** | **Time (2 h)** | **Time (3 h)** | **Average^a^** |
| --- | --- | --- | --- | --- | --- | --- |
| *-* | *-* | *-* | 0.73 | 0.82 | 0.87 | ***0.81 (0.54, 1.08)*** |
| *-* | *-* | *+* | 0.95 | 1.06 | 1.15 | ***1.06 (0.79, 1.32)*** |
| *+* | *-* | *-* | 0.64 | 0.72 | 0.76 | *0.71 (0.44, 0.97)* |
| *+* | *-* | *+* | 1.18 | 1.34 | 1.45 | *1.32 (1.05, 1.59)* |
| *-* | *+* | *-* | 0.62 | 0.69 | 1.05 | **0.79 (0.52, 1.06)** |
| *-* | *+* | *+* | 1.26 | 1.42 | 1.55 | **1.41 (1.14, 1.68)*** |
| *+* | *+* | *-* | 0.77 | 0.90 | 1.00 | 0.89 (0.62, 1.16) |
| *+* | *+* | *+* | 1.67 | 1.90 | 2.10 | 1.89 (1.62, 2.16) |
|  |  |  |  |  |  |  |
| **Effect size of MetS and H_2_O_2_ interaction (P_interaction_ = 0.049)** | | | | | | |
| MetS | 0.08 (95% CI: -0.18, 0.35) | | | | | |
| H_2_O_2_ | 0.43 (95% CI: 0.17, 0.70) | | | | | |
| Interaction | 0.46 (95%CI: 0.20, 0.73) | | | | | |
| **Effect size of VA and H_2_O_2_ interaction (P_interaction_ = 0.053)^b^** | | | | | | |
| VA | -0.01 (95% CI: -0.21, 0.27) | | | | | |
| H_2_O_2_ | 0.44 (95% CI: 0.17, 0.70) | | | | | |
| Interaction | 0.37 (95%CI: 0.11, 0.64) | | | | | |

^a^Values are mean (95% confidence interval) of intracellular ROS production in fluorescence units, adjusted for time. ^b^Estimation of effect size is based on host-hoc test; this is statistically significant, although it should be emphasized that the overall ANOVA only showed a difference of borderline statistical significance for the interaction. Bold text cover groups involved in the MetS and H_2_O_2_ interaction. Italic text cover groups involved in the FA and H_2_O_2_ interaction.

**Table S6.** Intracellular ROS production in cells after exposure to 5 µM VA

| **VA (5 µM)** | **MetS** | **H_2_O_2_** | **Time (1 h)** | **Time (2 h)** | **Time (3 h)** | **Average^a^** |
| --- | --- | --- | --- | --- | --- | --- |
| *-* | *-* | *-* | 0.74 | 0.82 | 0.90 | ***0.82 (0.54, 1.11)*** |
| *-* | *-* | *+* | 0.93 | 1.04 | 1.13 | ***1.03 (0.75, 1.32)*** |
| *+* | *-* | *-* | 0.69 | 0.79 | 0.86 | *0.78 (0.49, 1.06)* |
| *+* | *-* | *+* | 1.33 | 1.52 | 1.66 | *1.50 (1.22, 1.78)*** |
| *-* | *+* | *-* | 0.80 | 0.88 | 0.96 | **0.88 (0.59, 1.62)** |
| *-* | *+* | *+* | 1.48 | 1.70 | 1.86 | **1.68 (1.40, 1.96)**** |
| *+* | *+* | *-* | 0.74 | 0.85 | 0.95 | 0.85 (0.56, 1.13) |
| *+* | *+* | *+* | 2.02 | 2.30 | 2.58 | 2.30 (2.02, 2.58) |
|  |  |  |  |  |  |  |
| **Effect size of MetS and H_2_O_2_ interaction (P_interaction_ = 0.001)** | | | | | | |
| MetS | 0.06 (95% CI: -0.22, 0.34) | | | | | |
| H_2_O_2_ | 0.47 (95% CI: 0.19, 0.75) | | | | | |
| Interaction | 0.72 (95%CI: 0.44, 1.00) | | | | | |
| **Effect size of VA and H_2_O_2_ interaction (P_interaction_ = 0.005)** | | | | | | |
| VA | -0.04 (95% CI: -0.32, 0.24) | | | | | |
| H_2_O_2_ | 0.51 (95% CI: 0.23, 0.79) | | | | | |
| Interaction | 0.54 (95%CI: 0.26, 0.82) | | | | | |

^a^Values are mean (95% confidence interval) of intracellular ROS production in fluorescence units, adjusted for time. Bold text cover groups involved in the MetS and H_2_O_2_ interaction. Italic text cover groups involved in the VA and H_2_O_2_ interaction.

**Table S7.** Intracellular ROS production in cells after exposure to 5 µM HA

| **HA (5 µM)** | **MetS** | **H_2_O_2_** | **Time (1 h)** | **Time (2 h)** | **Time (3 h)** | **Average^a^** |
| --- | --- | --- | --- | --- | --- | --- |
| *-* | *-* | *-* | 1.00 | 1.10 | 1.17 | ***1.09 (0.81, 1.37)*** |
| *-* | *-* | *+* | 1.08 | 1.22 | 1.31 | ***1.20 (0.92, 1.49)*** |
| *+* | *-* | *-* | 0.66 | 0.74 | 0.80 | *0.73 (0.45, 1.02)* |
| *+* | *-* | *+* | 1.29 | 1.49 | 1.57 | *1.45 (1.17, 1.73)*** |
| *-* | *+* | *-* | 0.75 | 0.82 | 0.88 | **0.82 (0.53, 1.10)** |
| *-* | *+* | *+* | 1.46 | 1.66 | 1.79 | **1.63 1.35, 1.92)***** |
| *+* | *+* | *-* | 0.61 | 0.72 | 0.79 | 0.70 (0.42, 0.99) |
| *+* | *+* | *+* | 1.92 | 2.19 | 2.38 | 2.16 (1.88, 2.45) |
|  |  |  |  |  |  |  |
| **Effect size of MetS and H_2_O_2_ interaction (P_interaction_ = 0.003)** | | | | | | |
| MetS | -0.15 (95% CI: -0.43, 0.13) | | | | | |
| H_2_O_2_ | 0.42 (95% CI: 0.13, 0.70) | | | | | |
| Interaction | 0.57 (95%CI: 0.29, 0.85) | | | | | |
| **Effect size of HA and H_2_O_2_ interaction (P_interaction_ < 0.001)** | | | | | | |
| HA | -0.23 (95% CI: -0.51, 0.05) | | | | | |
| H_2_O_2_ | 0.46 (95% CI: 0.18, 0.75) | | | | | |
| Interaction | 0.39 (95%CI: 0.10, 0.69) | | | | | |

^a^Values are mean (95% confidence interval) of intracellular ROS production in fluorescence units, adjusted for time. Bold text cover groups involved in the MetS and H_2_O_2_ interaction. Italic text cover groups involved in the HA and H_2_O_2_ interaction.

**Table S8.** Intracellular ROS production in cells after exposure to 50 µM HA

| **HA (50 µM)** | **MetS** | **H_2_O_2_** | **Time (1 h)** | **Time (2 h)** | **Time (3 h)** | **Average^a^** |
| --- | --- | --- | --- | --- | --- | --- |
| *-* | *-* | *-* | 0.93 | 1.05 | 1.13 | 1.04 (0.76, 1.31) |
| *-* | *-* | *+* | 1.43 | 1.58 | 1.70 | **1.57 (1.30, 1.84)^a^** |
| *+* | *-* | *-* | 0.78 | 0.97 | 1.05 | 0.93 (0.66, 1.20) |
| *+* | *-* | *+* | 1.59 | 1.65 | 1.79 | **1.68 (1.40, 1.95)^a^** |
| *-* | *+* | *-* | 0.72 | 0.92 | 0.99 | 0.88 (0.61, 1.15) |
| *-* | *+* | *+* | 1.21 | 1.49 | 1.62 | **1.44 (1.17, 1.71)^a^** |
| *+* | *+* | *-* | 0.78 | 0.90 | 0.98 | 0.89 (0.62, 1.16) |
| *+* | *+* | *+* | 1.59 | 1.85 | 2.02 | **1.82 (1.55, 2.09)^a^** |
|  |  |  |  |  |  |  |
| **Effect size of MetS and H_2_O_2_ interaction (P_interaction_ = 0.58)** | | | | | | |
| MetS | -0.10 (95% CI: -0.37, 0.17) | | | | | |
| H_2_O_2_ | 0.64 (95% CI: 0.37, 0.91) | | | | | |
| Interaction | 0.01 (95% CI: -0.26, 0.28) | | | | | |
| **Effect size of HA and H_2_O_2_ interaction (P_interaction_ = 0.13)** | | | | | | |
| HA | -0.05 (95% CI: -0.32, 0.22) | | | | | |
| H_2_O_2_ | 0.55 (95% CI: 0.28, 0.82) | | | | | |
| Interaction | 0.24 (95% CI: -0.03, 0.51) | | | | | |

^a^Values are mean (95% confidence interval) of intracellular ROS production in fluorescence units, adjusted for time. Bold text cover groups with single-factor effect of H_2_O_2_.

**Table S9.** Intracellular ROS production in cells after exposure to 6.1 µM Mix

| **Mix (6.1 µM)** | **MetS** | **H_2_O_2_** | **Time (1 h)** | **Time (2 h)** | **Time (3 h)** | **Average (95% CI)^a^** |
| --- | --- | --- | --- | --- | --- | --- |
| *-* | *-* | *-* | 0.95 | 1.07 | 1.18 | ***1.07 (0.83, 1.30)*** |
| *-* | *-* | *+* | 1.31 | 1.49 | 1.59 | ***1.46 (1.23, 1.70)*** |
| *+* | *-* | *-* | 0.83 | 0.90 | 0.98 | *0.90 (0.66, 1.37)* |
| *+* | *-* | *+* | 1.67 | 1.92 | 2.01 | *1.89 (1.65, 2.12)*** |
| *-* | *+* | *-* | 0.85 | 0.95 | 1.01 | **0.94 (0.70, 1.17)** |
| *-* | *+* | *+* | 1.64 | 1.88 | 2.02 | **1.84 (1.61, 2.08)*** |
| *+* | *+* | *-* | 0.94 | 1.03 | 1.11 | 1.02 (0.79, 1.26) |
| *+* | *+* | *+* | 2.03 | 2.30 | 2.56 | 2.30 (2.07, 5.53) |
|  |  |  |  |  |  |  |
| **Effect size of MetS and H_2_O_2_ interaction (P_interaction_ = 0.019)** | | | | | | |
| MetS | -0.00 (95% CI: -0.24, 0.23) | | | | | |
| H_2_O_2_ | 0.69 (95% CI: 0.46, 0.93) | | | | | |
| Interaction | 0.40 (95%CI: 0.16, 0.63) | | | | | |
| **Effect size of Mix and H_2_O_2_ interaction (P_interaction_ = 0.006)** | | | | | | |
| Mix | -0.04 (95% CI: -0.28, 0.20) | | | | | |
| H_2_O_2_ | 0.65 (95% CI: 0.42, 0.89) | | | | | |
| Interaction | 0.44 (95%CI: 0.20, 0.67) | | | | | |

^a^Values are mean (95% confidence interval) of intracellular ROS production in fluorescence units, adjusted for time. Bold text cover groups involved in the MetS and H_2_O_2_ interaction. Values in Italic text cover groups involved in the Mix and H_2_O_2_ interaction.

**
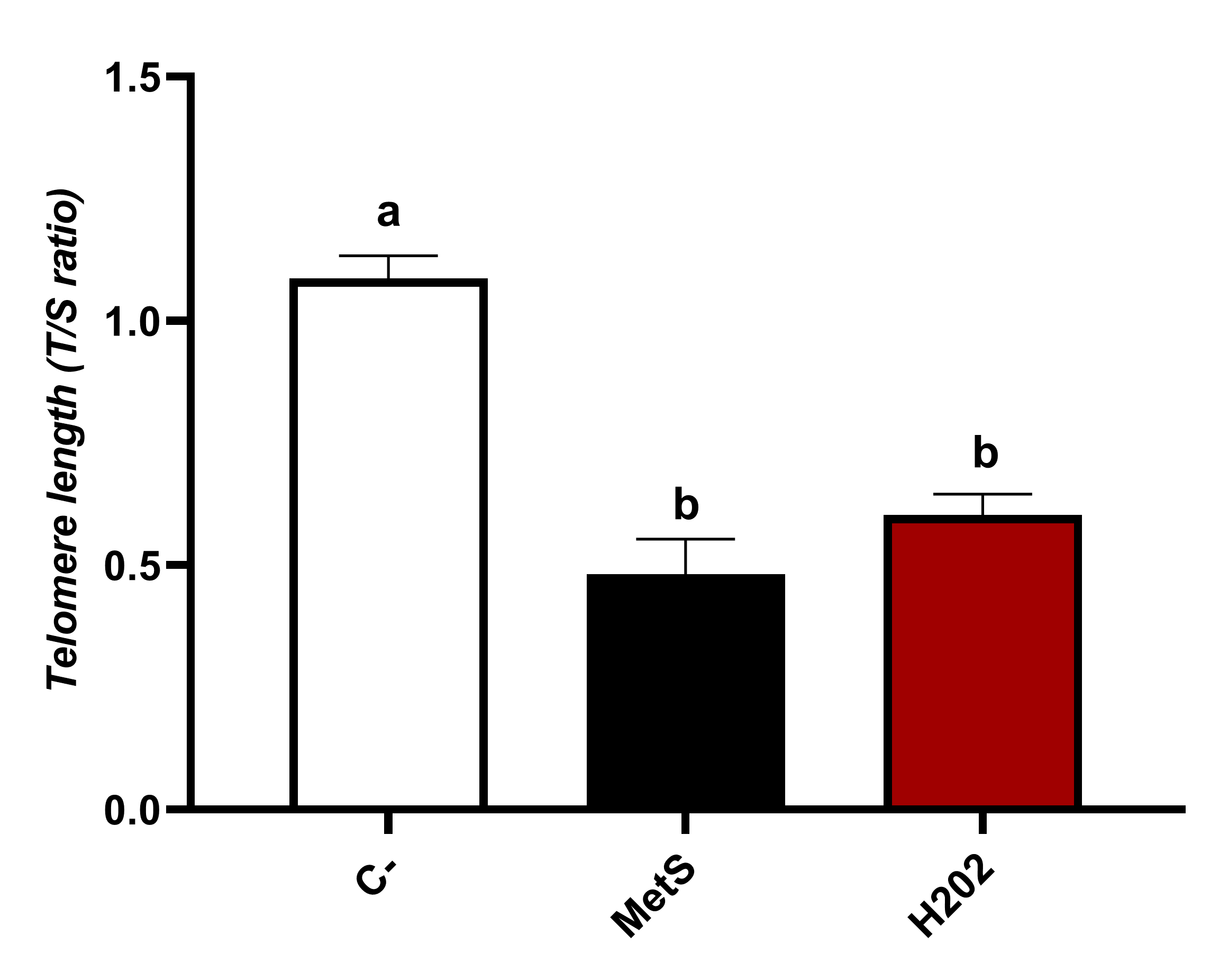
**

**Figure S1:** Telomere length (TL) in THP-1 cells after 72 h exposure to MetS stimulus or H₂O₂. Cells were treated with a MetS mixture consisting of free fatty acids (FFAs; oleic acid:palmitic acid in a 2:1 ratio, 500 μM) combined with TNF-α (1 ng/mL), or with hydrogen peroxide (H₂O₂, 200 nM) as a positive control. Untreated control cells received vehicles only (2% BSA and 0.05% methanol). TL was measured by quantitative real-time PCR and expressed as relative T/S ratio normalized to the control. Data are presented as mean ± SEM (n = 3). Significant differences between groups are indicated by different letters (a, b; p < 0.001).
